# Rationale and design of two randomized sham-controlled trials of catheter-based renal denervation in subjects with uncontrolled hypertension in the absence (SPYRAL HTN-OFF MED Pivotal) and presence (SPYRAL HTN-ON MED Expansion) of antihypertensive medications: a novel approach using Bayesian design

**DOI:** 10.1007/s00392-020-01595-z

**Published:** 2020-02-07

**Authors:** Michael Böhm, Raymond R. Townsend, Kazuomi Kario, David Kandzari, Felix Mahfoud, Michael A. Weber, Roland E. Schmieder, Konstantinos Tsioufis, Graeme L. Hickey, Martin Fahy, Vanessa DeBruin, Sandeep Brar, Stuart Pocock

**Affiliations:** 1 Universitätsklinikum des Saarlandes, Saarland University, Homburg, Germany; 2 Perelman School of Medicine, University of Pennsylvania, Philadelphia, PA USA; 3 Jichi Medical University, Tochigi, Japan; 4 Piedmont Heart Institute, Atlanta, GA USA; 5 State University of New York, Downstate Medical Center, Brooklyn, NY USA; 6 University Hospital Erlangen, Erlangen, Germany; 7 National and Kapodistrian University of Athens, Athens, Greece; 8 Medtronic PLC, Santa Rosa, CA USA; 9 London School of Hygiene and Tropical Medicine, London, UK

**Keywords:** Blood pressure, Device-based statistics, Renal sympathetic nervous system, Sham, Trial design

## Abstract

**Background:**

The SPYRAL HTN clinical trial program was initiated with two 80-patient pilot studies, SPYRAL HTN-OFF MED and SPYRAL HTN-ON MED, which provided biological proof of principle that renal denervation has a blood pressure-lowering effect versus sham controls for subjects with uncontrolled hypertension in the absence or presence of antihypertensive medications, respectively.

**Trial design:**

Two multicenter, prospective, randomized, sham-controlled trials have been designed to evaluate the safety and efficacy of catheter-based renal denervation for the reduction of blood pressure in subjects with hypertension in the absence (SPYRAL HTN-OFF MED Pivotal) or presence (SPYRAL HTN-ON MED Expansion) of antihypertensive medications. The primary efficacy endpoint is baseline-adjusted change from baseline in 24-h ambulatory systolic blood pressure. The primary safety endpoint is incidence of major adverse events at 1 month after randomization (or 6 months in cases of new renal artery stenosis). Both trials utilize a Bayesian design to allow for prespecified interim analyses to take place, and thus, the final sample sizes are dependent on whether enrollment is stopped at the first or second interim analysis. SPYRAL HTN-OFF MED Pivotal will enroll up to 300 subjects and SPYRAL HTN-ON MED Expansion will enroll up to 221 subjects. A novel Bayesian power prior approach will leverage historical information from the pilot studies, with a degree of discounting determined by the level of agreement with data from the prospectively powered studies.

**Conclusions:**

The Bayesian paradigm represents a novel and promising approach in device-based hypertension trials.

**Clinical trial registration:**

URL: https://www.clinicaltrials.gov. Unique identifier: NCT02439749 (SPYRAL HTN-OFF MED Pivotal) and NCT02439775 (SPYRAL HTN-ON MED Expansion).

**Electronic supplementary material:**

The online version of this article (10.1007/s00392-020-01595-z) contains supplementary material, which is available to authorized users.

## Introduction

Catheter-based renal denervation has been shown to decrease central sympathetic activity, with early clinical trials showing blood pressure-lowering effects in subjects with uncontrolled hypertension [1, 2]. Despite these early findings, the randomized, blinded, sham-controlled SYMPLICTY HTN-3 trial showed a statistically significant reduction from baseline in blood pressure in both the renal denervation and sham control groups, but the difference between the two groups was not significant [3]. A post hoc analysis showed that lack of adherence to antihypertensive medication regimens, and fewer ablations during the denervation procedure were associated with decreased systolic blood pressure (SBP) effects in this trial [4].

The SPYRAL HTN clinical trial program was initiated with pilot studies that were designed to control for the potentially confounding effects of the variables identified in the post hoc analysis. Separate pilot studies, SPYRAL HTN-OFF MED and SPYRAL HTN-ON MED, reaffirmed the biological proof of principle that renal denervation had a significant blood pressure-lowering effect versus sham controls for subjects with uncontrolled hypertension in the absence or presence of antihypertensive medications, respectively [5, 6]. An important difference from previous studies was use of a redesigned catheter and treatment algorithms that allow for more distal circumferential treatment of the main and branch vessels.

To transition seamlessly into the next stage of the program, the prospectively powered SPYRAL HTN-OFF MED Pivotal trial and SPYRAL HTN-ON MED Expansion trial will leverage information from the pilot studies, which share a similar design. This is achieved with the use of a novel Bayesian design that leverages information from these pilot studies, and applies a discount function that adjusts the influence of the historical information based on level of agreement with the data from the prospectively powered studies [7–9]. Additionally, the design incorporates prespecified interim analyses. Such approaches, particularly in the Bayesian paradigm, have become adopted in recent clinical trials and have shown great promise [10, 11]. This approach allows for the same rigor as the Frequentist approach but with more efficient use of data, faster timelines, and reduced cost [12]. In this report, we describe the use of a unique version of this design in the SPYRAL HTN-OFF MED Pivotal and SPYRAL HTN-ON MED Expansion trials.

## Methods

### Program overview

Two multicenter, international, prospective, single blinded, randomized, sham-controlled trials in the SPYRAL HTN clinical trial program are evaluating the safety and efficacy of catheter-based renal denervation for the reduction of blood pressure in subjects with uncontrolled hypertension. The SPYRAL HTN-OFF MED Pivotal trial is evaluating catheter-based renal denervation for the reduction of blood pressure in the absence of antihypertensive medications, and the SPYRAL HTN-ON MED Expansion trial in the presence of 1–3 guideline-recommended antihypertensive medications. In each trial, subjects randomized to the renal denervation group undergo the renal denervation procedure, and those randomized to the control group receive a sham procedure, with the subject remaining on the catheterization table for at least 20 min before sheath removal.

The SPYRAL HTN-OFF MED Pivotal and SPYRAL HTN-ON MED Expansion trials are sponsored by Medtronic (Santa Rosa, CA, USA), and were designed by the principal investigators, steering committees, and sponsor in collaboration with the FDA and followed recommendations provided by European and US consensus documents [13, 14]. The trials are being conducted in accordance with the Declaration of Helsinki 2013, the international standard ISO 14155:2011, and local laws and regulations, including data protection laws. The study protocols were approved by the institutional review boards and/or ethics committees at each clinical site, and written informed consent is provided by all subjects before enrollment. The trials are registered at ClinicalTrials.gov with the identifiers NCT02439749 (SPYRAL HTN-OFF MED Pivotal) and NCT02439775 (SPYRAL HTN-ON MED Expansion).

### Trial design

The SPYRAL HTN-OFF MED Pivotal and SPYRAL HTN-ON MED Expansion trials will each include 80 subjects from the respective pilot studies for use as an informative prior (see section on “Statistics”, “Determination of sample size and prespecified interim looks”). For SPYRAL HTN-OFF MED Pivotal, additional 247–353 subjects will be randomized for an overall sample size up to 433 subjects. For SPYRAL HTN-ON MED Expansion, additional 175–260 subjects will be randomized for an overall sample size up to 340 subjects. For both trials, subjects are being enrolled in study sites across the United States, Canada, Japan, Australia, and countries where the CE mark applies (up to 50 sites for SPYRAL HTN-OFF MED Pivotal and up to 55 sites for SPYRAL HTN-ON MED Expansion).

Eligible subjects are 20–80 years old and have hypertension, defined as office systolic blood pressure (SBP) ≥ 150 mm Hg and < 180 mm Hg, office diastolic blood pressure (DBP) ≥ 90 mm Hg, and mean 24 h SBP ≥ 140 mm Hg and < 170 mm Hg. For the SPYRAL HTN-OFF MED Pivotal trial, subjects must be naïve to antihypertensive medications or willing to discontinue all antihypertensive medications from the first screening visit through assessment of the primary efficacy endpoint (3 months post-procedure). For the SPYRAL HTN-ON MED Expansion trial, subjects must be taking 1–3 antihypertensive medications prescribed at ≥ 50% of the maximum manufacturer’s dosage. Antihypertensive medication classes must include a thiazide-type diuretic, a dihydropyridine calcium channel blocker, an angiotensin-converting enzyme-I/angiotensin-II receptor blocker, and/or a beta-blocker. Subjects must be on a stable dose of each medication for at least 6 weeks before the first screening visit and continuing until a confirmatory second screening visit. When prescribed other qualifying medications, 12.5 mg hydrochlorothiazide is acceptable as the minimum dosage. A complete list of inclusion and exclusion criteria are presented in Table 1.

**Table 1:** Inclusion and exclusion criteria for the SPYRAL HTN-OFF MED Pivotal and SPYRAL HTN-ON MED Expansion trials

**Inclusion criteria**
Age 20–80 years at enrolment
Office SBP < 180 mm Hg^a,b^
Office SBP ≥ 150 mm Hg^a,c^
Office DBP ≥ 90 mm Hg^a,c^
24 h ABPM average SBP ≥ 140 mm Hg and < 170 mm Hg^d^^−^^f^
SPYRAL HTN-OFF MED Pivotal only: willing to discontinue current antihypertensive medications at Screening Visit 1 through the 3-month post-procedure visit
Agrees to have all study procedures performed, is competent and willing to provide written informed consent
**Exclusion criteria**
Undergone prior renal denervation
Renal anatomy that is ineligible for treatment
Main renal artery for each kidney is < 3 mm or > 8 mm
Lack of main renal arterial vessel that does not allow four simultaneous quadratic ablations in main renal artery or equivalent
Presence of fibromuscular dysplasia
> 50% stenosis in any treatable vessel
Renal artery stent placed < 3 months prior to denervation procedure
Presence of aneurysm defined as any localized increase in vessel diameter
Treatment area within 5 mm segment in the renal artery contains an atheroma, calcification, or renal artery stent
eGFR < 45 mL/min/1.73 m^2^, using the 4-variable MDRD calculation^g^
Taking SGLT2 inhibitors or GLP-1 agonists that have been prescribed < 90 days prior to Screening Visit 1 or without plan to remain on those medications for duration of trial
≥ 1 episode of orthostatic hypotension not related to medication changes within the past year or a reduction in SBP ≥ 20 mm Hg or DBP ≥ 10 mm Hg within 3 min of standing coupled with symptoms during the screening process
Requires chronic oxygen support or mechanical ventilation (other than nocturnal respiratory support for sleep apnea)
Documented primary pulmonary hypertension
Untreated secondary cause of hypertension (known or suspected) or taking medications that increase sympathetic tone that could contribute to hypertension
Frequent intermittent or chronic pain that results in treatment with NSAIDs for ≥ 2 days per week over the month prior to enrollment (aspirin and clopidogrel permitted for cardiovascular risk reduction)
HIV on anti-retroviral drug therapy but without documentation that hypertension preceded initiation of anti-retroviral drug therapy
SPYRAL HTN-OFF MED Pivotal: history of myocardial infarction, stable or unstable angina, transient ischemic attack, cerebrovascular accident, heart failure, or atrial fibrillation within 3 months of enrolment
SPYRAL HTN-ON MED Expansion: History of myocardial infarction, unstable angina pectoris, syncope, transient ischemic attack, or a cerebrovascular accident within 3 months of the screening period, or widespread atherosclerosis with documented intravascular thrombosis or unstable plaques
Peptic ulcer or gastrointestinal bleeding within 6 months before consent
History of bleeding diathesis or coagulopathy or refuses blood transfusions
Polycystic kidney disease, unilateral kidney, or history of renal transplant
Scheduled or planned surgery that may affect study endpoints, in opinion of Investigator
Documented condition that would prohibit or interfere with ability to obtain an accurate blood pressure measurement (e.g., upper arm circumference outside cuff size ranges available by geography or arrhythmia that interferes with automatic monitor’s pulse sensing)
Severe cardiac valve stenosis for which a significant reduction of blood pressure is contraindicated, in opinion of investigator
Documented confounding medical condition that may adversely affect the safety of the subject, in opinion of investigator (e.g., clinically significant peripheral vascular disease or aortic aneurysm)
Currently prescribed narcotic drugs or methadone
Currently taking anti-mineralocorticoid medications, unless weaned off by ≥ 8 weeks prior to Screening Visit 1
Known unresolved history of drug use or alcohol dependency, lacks ability to comprehend or follow instructions, or would be unlikely or unable to comply with study follow-up requirements
Currently enrolled in a concurrent investigational drug or device study, unless approved by study sponsor
Pregnant, nursing or planning to become pregnant during course of the study or follow-up
Works night shifts

*ABPM* ambulatory blood pressure monitoring, *DBP* diastolic blood pressure, *eGFR* estimated glomerular filtration rate, *MDRD* modification of diet in renal disease, *SBP* systolic blood pressure

^a^For SPYRAL HTN-ON MED Expansion, criterion applies when subject is on 1–3 antihypertensive medications at ≥ 50% of the maximum manufacturer’s dosage. Antihypertensive medication classes must include a thiazide-type diuretic, a dihydropyridine calcium channel blocker, an angiotensin-converting enzyme-I/angiotensin-II receptor blocker, or a beta-blocker. Subject must be on a stable dose of each medication for at least 6 weeks before Screening Visit 1 and up to Screening Visit 2. When prescribed other qualifying medications, 12.5 mg hydrochlorothiazide is acceptable as the minimum dosage. In Japan, subjects may be prescribed less than 50% of the maximum manufacturer’s recommended dosage of a thiazide-type diuretic, per standard of care

^b^Applied at Screening Visits 1 and 2 for both trials

^c^Applied at Screening Visit 2 for HTN-OFF MED, Screening Visits 1 and 2 for SPYRAL HTN-ON MED Expansion

^d^Applied at Screening Visit 2 for both trials

^e^ABPM is considered valid if the number of successful daytime readings captured is ≥ 21 and the number of successful nighttime readings captured is ≥ 12

^f^For SPYRAL HTN-ON MED Expansion, ABPM device is applied after witnessed ingestion of antihypertensive medications

^g^eGFR calculation specific to Japanese subjects will be used for subjects enrolled in Japan

**Fig. 1:**
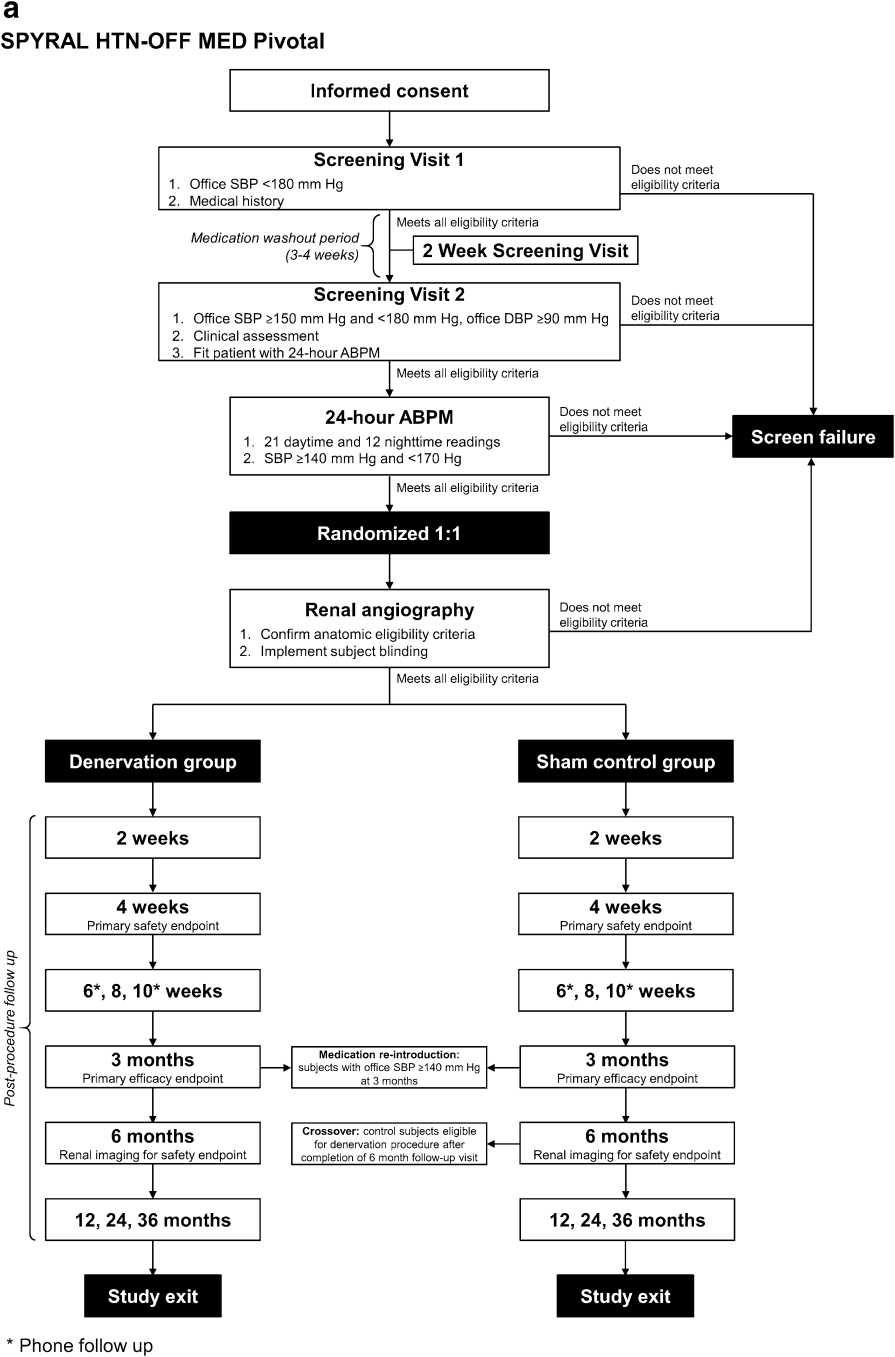

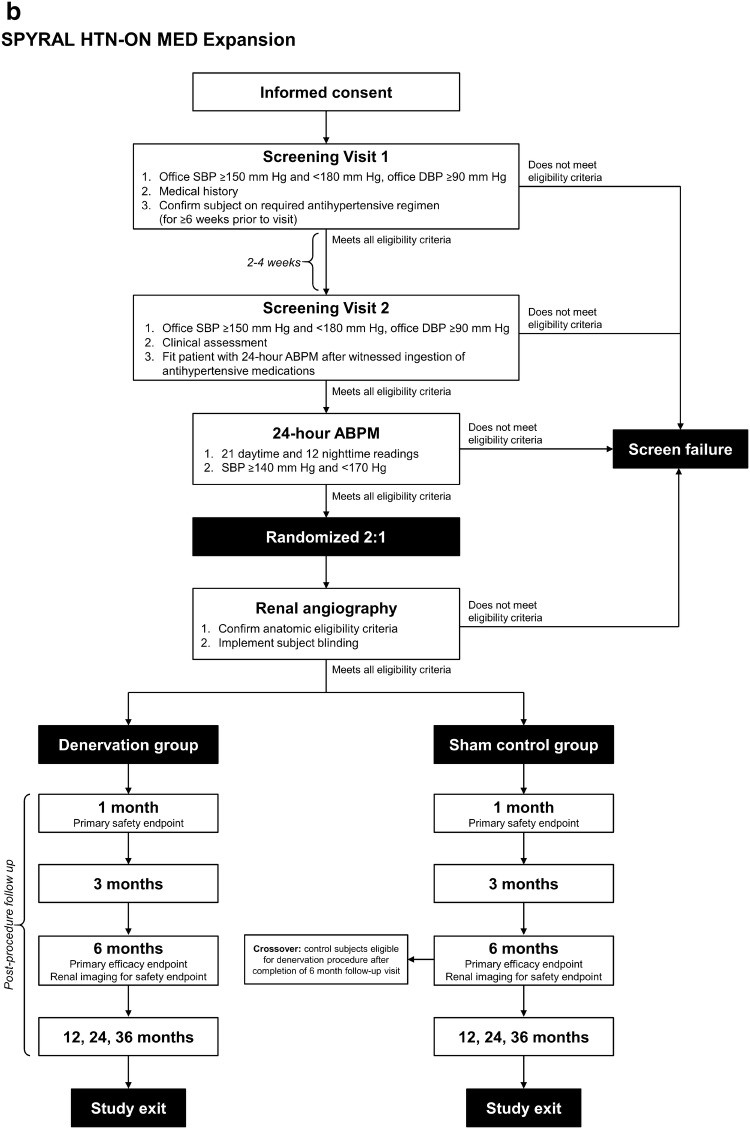
Subject flow through the SPYRAL HTN-OFF MED Pivotal (**a**) and SPYRAL HTN-ON MED Expansion (**b**) trials; *ABPM* ambulatory blood pressure monitor, *DBP* diastolic blood pressure, *SBP* systolic blood pressure

The details of each trial design are shown in Fig. 1. Subjects undergo a series of screening visits before randomization into treatment groups. At the first visit (Screening Visit 1), subjects are screened based on medical history and office SBP. After a continued medication stabilization period of 2–4 weeks (SPYRAL HTN-ON MED Expansion) or medication-washout period of up to 4 weeks (SPYRAL HTN-OFF MED Pivotal), eligible subjects proceed to a second visit (Screening Visit 2) where they are screened for potential randomization based on office BP and clinical assessment. Subjects who continue to meet eligibility criteria at this visit are fitted with a 24 h ABPM device as a final screen before randomization. Baseline BP measurements are obtained at Screening Visit 2.

Subjects who meet the 24 h ambulatory SBP criteria proceed to renal angiography to confirm that renal anatomy meets eligibility criteria. Upon confirmation of final eligibility criteria, subjects are randomized into treatment groups (1:1 denervation to control for SPYRAL HTN-OFF MED Pivotal, 2:1 denervation to control for SPYRAL HTN-ON MED Expansion). Subjects are blinded during renal angiography as to what treatment procedure (RDN or sham) they received and, following the angiogram, those randomized to the renal denervation group proceed directly to the renal artery denervation procedure. To ensure potential contributory factors other than the renal denervation procedure are controlled for, a sham control, as included in the SYMPLICITY HTN-3 trial, is included in both trials. As such, those in the control group receive a sham procedure, where the subject remains on the catheterization lab table for at least 20 min prior to sheath removal. All subjects receive conscious sedation to ensure proper blinding.

For SPYRAL HTN-OFF MED Pivotal, subjects return for office follow-up visits at 2, 4, and 8 (± 3 days) weeks, 3 and 6 (± 14 days) months, and 12, 24, and 36 (± 30 days) months post-procedure. Phone follow-ups are also conducted at 6 and 10 (± 3 days) weeks. Upon completion of the 3-month visit, subjects with office SBP ≥ 140 mm Hg will receive antihypertensive medications according to a medication re-introduction protocol (supplement). For SPYRAL HTN-ON MED Expansion, subjects return for office follow-up visits at 1, 3, and 6 (± 14 days) months and 12, 24, and 36 (± 30 days) months post-procedure. Antihypertensive medication changes are not allowed through 6 months in the SPRYAL HTN-ON MED Expansion trial.

Each trial also has an escape protocol for subjects who need to adjust antihypertensive medications after randomization. Subjects who met antihypertensive medication escape criteria will have blood pressure measurements analyzed with Last Observation Carried Forward (LOCF, see section on “Statistics”, “Analysis populations”). For both trials, if a subject has office SBP ≥ 180 mm Hg or there is a safety concern between randomization and 3 months (SPYRAL HTN-OFF MED Pivotal) or 6 months (SPYRAL HTN-ON MED Expansion) post-procedure, the subject will be seen a second time within 72 h for a repeat office SBP. If the subject’s office SBP remains ≥ 180 mm Hg, antihypertensive medications will be reinstated per the investigator’s discretion. For SPYRAL HTN-ON MED Expansion, if a subject has office SBP < 115 mm Hg associated with symptoms of hypotension, the antihypertensive medication regimen may be changed and/or other therapy administered per the investigator’s discretion.

In both trials, subjects in the control group may crossover to receive renal denervation after completing the 6-month follow-up visit. Required baseline data must be collected and the subject must not meet any anatomic or estimated glomerular filtration rate (eGFR) exclusion criteria to receive the crossover renal denervation procedure. After the procedure, crossover subjects will undergo follow-up visits at 1, 3, and 6 (± 14 days) months and 12 and 24 (± 30 days) months (also 36 months for SPYRAL HTN-OFF MED Pivotal). Subjects who do not meet eligibility criteria for the crossover procedure will continue follow-up visits according to the original schedule.

### Screening, randomization, and blinding

Screening details have been previously described for each trial [5, 6, 15]. Randomization is stratified by study center, and each site can access randomization allocation via a password-protected system that can only be accessed by those approved by the sponsor.

The investigator performing catheterization and designated study staff are blinded to a subject’s randomization up until renal angiography is completed and eligibility is confirmed. Subjects are blinded during renal angiography by a combination of conscious sedation, sensory isolation (e.g., blindfold and music), and lack of familiarity with the procedural details and duration. Each study site has designated staff that are blinded to subject treatment group and responsible for obtaining office blood pressure and ambulatory blood pressure measurements. Blinding effectiveness of the subject will be assessed at discharge and follow-up visits through the 6-month blinding period by asking blinded staff and the study subject which group they believe the subject was randomized to. The unblinded personnel will not see the subject until the scheduled unblinding visit was completed.

### Symplicity Spyral catheter

The Symplicity Spyral multi-electrode renal denervation system is composed of the single-use disposable Symplicity Spyral catheter and the reusable Symplicity G3™ radiofrequency generator. Together, these components deliver low-level radiofrequency energy through the wall of the renal artery to denervate the renal nerves. The Symplicity Spyral catheter and Symplicity G3 generator received CE Mark in October 2013 and have been commercially available in selected geographies outside the United States, Canada, and Japan. Details of the Symplicity catheter design have been previously described [15].

### Procedure

For subjects randomized to the denervation group, the renal artery denervation procedure is performed according to the Symplicity Spyral catheter Instructions for Use, Symplicity G3 generator User Manual, and associated training provided by the sponsor. Therefore, based on sponsor recommendations, ablations are to be performed in all accessible renal arterial branch vessels with diameters between 3 and 8 mm, including accessory, branch, and main renal arteries that are outside of the renal parenchyma. Initial placement of the Symplicity Spyral catheter should be just proximal to the renal parenchyma, using fluoroscopic imaging for guidance. The investigators are instructed to perform as many ablations within a segment as anatomy permits, starting distally and working proximally without overlapping treatment zones. If the vessel segment cannot accommodate all four electrodes, then it is recommended to position a smaller number of electrodes and deselect the electrodes that are outside of the target segment. Ablations should be avoided in bifurcations and within 5 mm of areas with calcification, atheroma, or stented lesions.

For subjects in either group, preprocedural treatment with anxiolytic and/or analgesic medications may be considered and is at the discretion of the investigator. Additional doses of anxiolytic and/or analgesic medications are permitted during the procedure to be timed with ablations as appropriate. Upon completion of the procedure, hemostasis at the puncture site should be achieved by manual compression or use of closure devices.

### Endpoints

The primary efficacy endpoint is baseline-adjusted change in 24 h ambulatory SBP from baseline to 3 months (SPYRAL HTN-OFF MED Pivotal) or 6 months (SPYRAL HTN-ON MED Expansion) post-procedure. SPYRAL HTN-OFF MED Pivotal includes a powered secondary efficacy endpoint, which is baseline-adjusted change in office SBP from baseline to 3 months. All endpoints, including secondary safety, efficacy, and quality of life measures, are summarized in Table 2. The primary safety endpoint for both trials is the 1-month incidence of major adverse events and occurrence of renal artery stenosis at 6 months following randomization. Major adverse events are defined as a composite of all-cause mortality, end-stage renal disease, significant embolic event resulting in end-organ damage, renal artery perforation or dissection requiring intervention, vascular complications, hospitalization for hypertensive crisis (not related to confirmed nonadherence with medications or the protocol), and new renal artery stenosis > 70% (confirmed by angiography and as determined by the angiography core laboratory). Details on blood pressure measurement methodology are provided in the Supplement.

**Table 2:** Primary and secondary endpoints of the SPYRAL HTN-OFF MED Pivotal and SPYRAL HTN-ON MED Expansion trials

Endpoint	Time after index procedure	
	SPYRAL HTN-OFF MED Pivotal	SPYRAL HTN-ON MED Expansion
**Powered primary safety endpoint**	1 month^b^	1 month^b^
Incidence of major adverse events^a^		
**Powered primary efficacy endpoint**	3 months	6 months
Baseline-adjusted change from baseline in SBP, measured by 24 h ABPM^c^		
**Powered secondary efficacy endpoint**	3 months	NA^d^
Baseline-adjusted change from baseline in office SBP		
**Secondary safety endpoints**	1, 3, 6, 12, 24, and 36 months	1, 3, 6, 12, 24, and 36 months
Significant embolic event resulting in end-organ damage^e^		
Renal artery perforation requiring intervention^e^		
Renal artery dissection requiring intervention^e^		
Vascular complications^e^		
All-cause mortality^f^		
End-stage renal disease		
≥ 40% decline in eGFR		
New myocardial infarction		
New stroke		
Renal artery reintervention		
Major bleeding according to TIMI definition^g^		
Increase in serum creatinine > 50% from Screening Visit 2		
New renal artery stenosis > 70%^h^		
Hospitalization for hypertensive crisis not related to confirmed non-adherence or the protocol		
**Composite safety secondary endpoint**^i^	3, 6, 12, 24, and 36 months	3, 6, 12, 24, and 36 months
**Secondary efficacy endpoints**	1, 3, 6, 12, 24, and 36 months	1, 3, 6, 12, 24, and 36 months
Change from baseline in SBP, by 24 h ABPM^c,j^		
Change from baseline in office SBP^c^		
Change from baseline in DBP, by 24 h ABPM^c,j^		
Change from baseline office in DBP^c^		
Incidence of achieving target office SBP (< 140 mm Hg)		
**Quality-of-life measures**		
EuroQol-5D		
SF-36^k^		

*ABPM* ambulatory blood pressure monitoring, *DBP* diastolic blood pressure, *eGFR* estimated glomerular filtration rate, *NA* not applicable, *SBP* systolic blood pressure, *SF-36* Short Form (36) Health Survey, *TIMI* thrombolysis in myocardial infarction

^a^Defined as composite of all-cause mortality, end-stage renal disease, significant embolic event resulting in end-organ damage, renal artery perforation or dissection requiring intervention, vascular complications, hospitalization for hypertensive crisis not related to confirmed non-adherence with medications or the protocol, and new renal artery stenosis > 70% (confirmed by angiography and as determined by the angiography core laboratory)

^b^1 month post-randomization or 6 months for new renal artery stenosis

^c^Change from baseline values acquired at Screening Visit 2

^d^SPYRAL HTN-ON MED Expansion trial does not include a powered secondary efficacy endpoint

^e^Procedural secondary safety endpoints compared between groups only at 1 month after index procedure in SPYRAL HTN-OFF MED Pivotal

^f^Chronic secondary safety endpoint compared between groups only at 3, 6, 12, 24, and 36 months after index procedure in SPYRAL HTN-OFF MED Pivotal and SPYRAL HTN-ON MED Expansion

^g^TIMI definition of major bleeding included intracranial hemorrhage, ≥ 5 g/dL decrease in hemoglobin concentration, ≥ 15% absolute decrease in hematocrit, or death due to bleeding within 7 days of procedure

^h^Confirmed by angiography and as determined by angiography core laboratory

^i^Composite safety secondary endpoint includes same events as those in the powered primary safety endpoint but assessed at 3, 6, 12, 24, and 36 months

^j^Compared between groups at 3, 6, 12, 24, and 36 months after index procedure

^k^SPYRAL HTN-OFF MED Pivotal only

### Safety and quality control

A Data Safety Monitoring Board (DSMB) has been established to monitor the health, safety, and welfare of subjects. The DSMB is composed of physicians with experience in clinical studies of hypertension and/or cardiovascular medicine and one biostatistician with experience in the analysis of clinical studies. The DSMB members are not investigators in the study and are independent of the sponsor. Prior to the first DSMB review, guidelines were established for the identification and evaluation of significant safety findings and/or increased frequency of events that might impact the rights, safety, or welfare of subjects. All materials, discussions, and proceedings of the DSMB are completely confidential. The proceedings of each DSMB are recorded in minutes. The DSMB Chairperson is responsible for providing a written recommendation regarding study conduct (e.g., continue as planned, specify a modification, or termination).

An independent Clinical Events Committee (CEC) has also been established to adjudicate any safety endpoints. The CEC is composed of physicians who have experience in clinical studies in hypertension and/or cardiovascular indications. The CEC members are not investigators in the study and are independent of the sponsor. Guidelines for the adverse event adjudication process were established at the first CEC meeting. Details on the definitions used for adjudication, the adjudication process, and the reporting of outcomes are provided in the CEC Charter. The proceedings of each CEC meeting are recorded in minutes.

All programming and endpoint analyses will be independently performed by Cytel Inc. (Cambridge, MA, USA).

### Statistics

#### Bayesian design and prespecified interim analyses

A Bayesian trial design was proposed that allows for prespecified interim analyses to take place in addition to leveraging pilot study data. For SPYRAL HTN-OFF MED Pivotal, assuming 15% attrition, interim analyses are expected to occur first term at approximately 210 and second-term when approximately 240 subjects have evaluable data, with a maximum sample size of 300 evaluable subjects if the trial does not stop at either the first or second interim look. The time from randomization of the first cohort to the second cohort and final cohort, if applicable, is lengthy due to stringent eligibility criteria and subsequent slow randomization rates. For SPYRAL HTN-ON MED Expansion, the expected sample sizes are 149 and 187 evaluable subjects, with a maximum sample size of 221 evaluable subjects. Actual numbers of evaluable subjects will be determined by the actual attrition, which is currently expected to be 15%. At each prespecified interim analysis, enrollment may be stopped for efficacy or expected futility. For SPYRAL HTN-OFF MED Pivotal, the primary and secondary efficacy endpoints will be evaluated during these prespecified interim looks, and enrollment will only stop at an interim analysis if both endpoints meet the following stopping criteria. For SPYRAL HTN-ON MED Expansion, the primary efficacy endpoint will be evaluated at prespecified interim looks. A distinction between the stopping for efficacy and futility is that the former is based solely on the observed/evaluable data, whereas the latter involves the observed/evaluable data, as well as imputation for subjects without evaluable data or not yet randomized.

The underlying model is a Bayesian analogue of the analysis of covariance (ANCOVA) model for SBP change from baseline, ${y}_{i}$, adjusted for baseline blood pressure and treatment arm. Due to a Bayesian power prior approach being used, a non-standard parameterization for the ANCOVA model is used to allow for informative prior distributions to be placed separately on the RDN and control arm effects:

$${y}_{i}= {\mu }_{t}I\left(i\in t\right)+{\mu }_{c}I\left(i\in c\right)+{x}_{i}\beta +{\varepsilon }_{i}; {\varepsilon }_{i} \sim N\left(0, {\sigma }^{2}\right),$$

where ${y}_{i}$ denotes the change from baseline in BP at follow-up, subscripts $i$ denote the $i$th subject with evaluable data, $I\left(i\in t\right)=1$ if subject $i$ is randomized to renal denervation, or $=0$ if randomized to sham control, and $\beta$ is the regression coefficient for the adjustment in mean-centered baseline BP, ${x}_{i}$. Similarly, $I\left(i\in c\right)=1$ if subject $i$ is randomized to sham control, and 0 otherwise. Letting $\mu ={\mu }_{t}-{\mu }_{c}$ denote the baseline-adjusted treatment effect of SBP change comparing renal denervation to sham control, at either prespecified interim (or final) look, the posterior probability of $\mu$ being less than zero is calculated. If this probability is $>0.975$, the trial is stopped, and efficacy declared. Type I error rates were calculated by extensive simulation under this interim analysis scheme (details below).

At each prespecified interim look, a test for futility is also made. In this case, the probability of futility is based on the maximum study size of evaluable subjects, which requires us to impute the outcomes for subjects who have not yet been enrolled. If the posterior probability of $\mu <0$ from this calculation is $<0.05$ for the primary efficacy endpoint (and secondary efficacy endpoint in the context of SPYRAL HTN-OFF MED Pivotal), then the study will have met the futility boundary and enrollment will be stopped. If the study stops for efficacy at either the first or second prespecified interim analysis, then any additional subjects that have been enrolled before the decision to stop has been made will not be part of the primary endpoint analysis, but will be combined with the interim analysis cohort and analyzed as a secondary efficacy analysis.

**Fig. 2:**
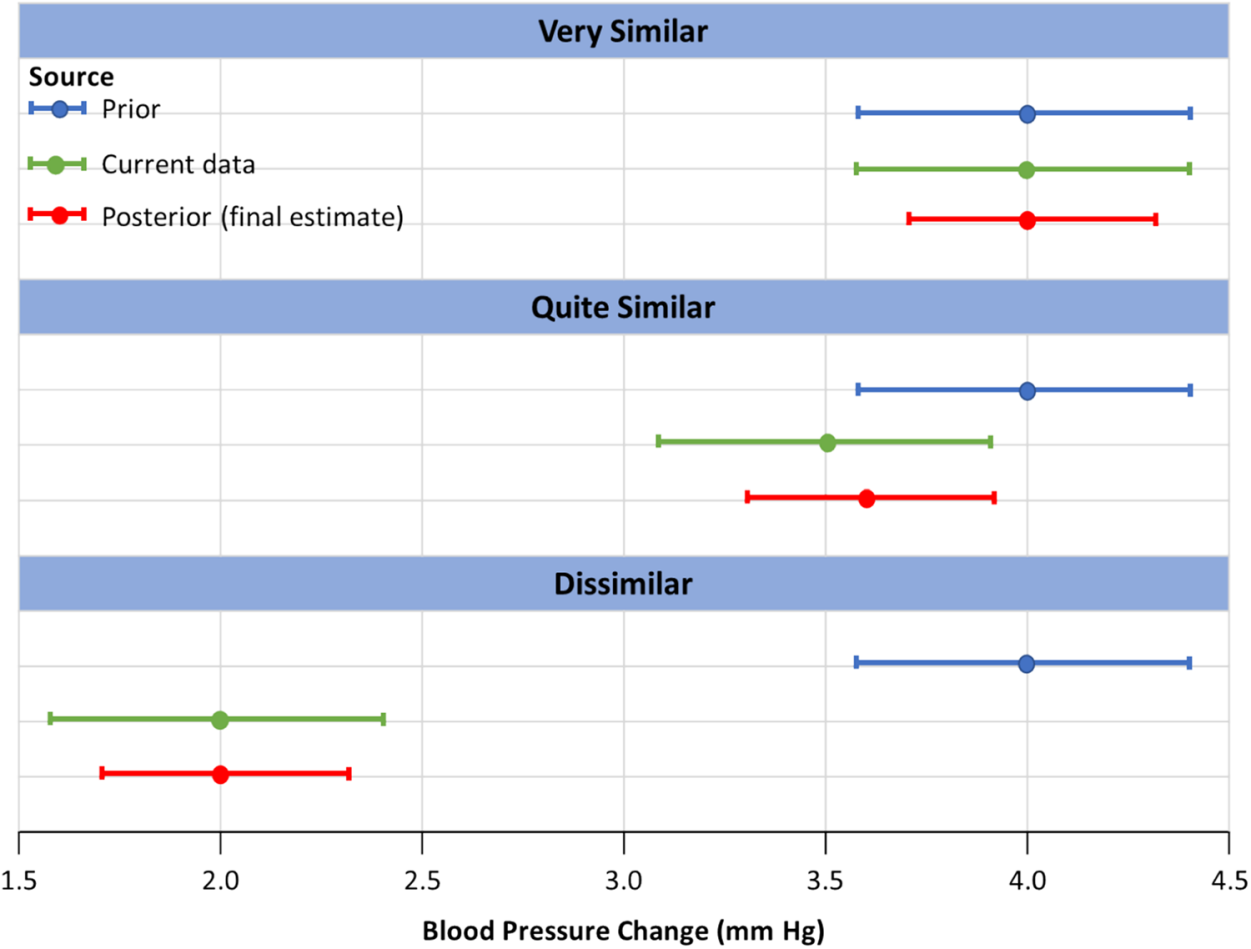
Diagrammatic illustration of the Bayesian discount prior methodology. Top panel: the prior data have similar outcomes to the current/pivotal data, meaning that nearly all information is utilized in the primary analysis. Middle panel: there is some overlap, meaning that the partial information is carried over into the primary analysis. Bottom panel: the outcomes are disparate, meaning that little-to-none data of the information from the prior are utilized for the primary analysis. Figure represents three hypothetical scenarios only

Since the pilot study data for SPYRAL HTN-OFF MED Pivotal and SPYRAL HTN-ON MED Expansion trials were collected under similar enrollment criteria and are published, these data are leveraged using a novel extension of the Bayesian power prior method, which was proposed by a Medical Device Innovative Consortium (MDIC) working group [5, 6, 16]. In brief, the first step involves updating flat prior distributions for ${\mu }_{t}$ and ${\mu }_{c}$ using the pilot study data only. These updated distributions are used as a prior distribution for analyzing the pivotal data. To allow for potential dissimilarities between the pilot studies and pivotal/prospective data, the aforementioned prior distributions will be down-weighted via separate power parameters, ${\alpha }_{\mathrm{t}}$ and ${\alpha }_{\mathrm{c}}$, respectively [9]. The power parameter can range between 0 (equivalent to ignoring all the pilot study data) and 1 (equivalent to complete pooling of the pilot study data). In addition, a diffuse normal prior distribution for $\beta$ and a flat prior on $\mathrm{l}\mathrm{o}\mathrm{g}(\sigma )$ are used to estimate the posterior distribution. A diagram illustrating the Bayesian discount prior methodology is provided in Fig. 2.

In practice, power parameters can be specified in advance or treated as random and estimated in the model. For this trial, we use a ‘dynamic borrowing’ approach based on the similarity of pilot study data to that of the pivotal/prospective data [17]. Namely, to determine the power parameters, a statistical comparison is first made between the pilot studies and pivotal/prospective data in each treatment arm following the approach described by Haddad and colleagues yielding comparison statistics ${p}_{t}$ and ${p}_{c}$, for the renal denervation and sham control arms, respectively [8]. A ‘*p* value’ close to 0 indicates a high probability that the pivotal/prospective data and pilot study data come from *different* populations, meaning that discounting should be applied to reduce the influence of the prior. Conversely, a *p* value close to 1 indicates that there is a high probability that the pivotal/prospective data and pilot study data come from similar populations and minimal discounting should be applied. The *p* values are transformed (also referred to as discounting) using a prespecified Weibull function with shape parameter $k=3$ and scale parameters $\lambda =0.25$ (SPYRAL HTN-ON MED Expansion) and 0.50 (SPYRAL HTN-OFF MED Pivotal); these functions were selected through tuning of the operating characteristics via extensive simulations.

### Determination of sample size and prespecified interim looks

For both the SPYRAL HTN-OFF MED Pivotal and SPYRAL HTN-ON MED Expansion cohorts, pilot study data are already available for 80 subjects. These data will be incorporated into the study via the aforementioned power prior methodology. As described earlier, sample sizes for SPYRAL HTN-OFF MED Pivotal and SPYRAL HTN-ON MED Expansion will vary due to the adaptive nature of the trial, and precise numbers of evaluable subjects will depend on the observed attrition rate. For SPYRAL HTN-OFF MED Pivotal and the first 26 randomized subjects in SPYRAL HTN-ON MED Expansion, subjects were randomized 1:1 to renal denervation or sham control. For subject 27 onwards in the SPYRAL HTN-ON MED Expansion study, randomization will be 2:1 for renal denervation to sham control following a protocol revision during the trial.

Each study includes prespecified interim analyses, at which point enrolment may be stopped for futility or expected success, as described previously. For SPYRAL HTN-OFF MED Pivotal, these will occur after the 210th and 240th subjects have reached their 3-month endpoint and have evaluable data, and for SPYRAL HTN-ON MED Expansion after the 175th and 220th subjects (excluding the 80 pilot study subjects) have reached their 6-month endpoint and have evaluable data. Enrolment will continue to the maximum study size of evaluable subjects if the study does not stop at either prespecified interim look.

In each study, the operating characteristics (including type I error and power) were determined using extensive simulations. A total of 8000 trial simulations were performed to estimate the power and 15,000 simulations to estimate the type I error. Interim look (and maximum) sample sizes as well as discount function parameters were tuned during simulation to achieve acceptable trial characteristics. Final study design parameter assumptions are described in Table 3. For SPYRAL HTN-OFF MED Pivotal, the overall trial power to detect a treatment difference of − 4.0 mm Hg in mean 24 h SBP was 94% with one-sided type I error 2.9% for the primary endpoint. Power at prespecified interim looks varied between 83 and 89%. For SPYRAL HTN-ON MED Expansion, the overall trial power to detect a treatment difference of − 5.0 mm Hg was 96% with type I error 3.0%. Power at first and second prespecified interim looks was 89% and 94%, respectively.

**Table 3:** Parameter assumptions for the SPYRAL HTN-OFF MED Pivotal and SPYRAL HTN-ON MED Expansion trials and summary of the available pilot study data used in the analysis

Simulation parameter	SPYRAL HTN-OFF MED Pivotal		SPYRAL HTN-ON MED Expansion
	Primary efficacy endpoint	Secondary efficacy endpoint	Primary efficacy endpoint
Pilot studies			
Pilot study treatment arm baseline-adjusted mean/SE	− 5.30/1.65 mm Hg	− 9.69/2.20 mm Hg	− 8.8/1.8 mm Hg
Pilot study treatment arm N	35	37	36
Pilot study control arm baseline-adjusted mean/SE	− 0.74/1.62 mm Hg	− 2.54/2.09 mm Hg	− 1.8/1.8 mm Hg
Pilot study control arm N	36	41	36
Maximum pilot study subjects	35 + 36 = 71	37 + 41 = 78	36 + 36 = 72
Pivotal study			
Pivotal/prospective study expected treatment difference	4.0 mm Hg	6.5 mm Hg	5.0 mm Hg
Pivotal/prospective study treatment arm mean/SD	− 4.74/12 mm Hg	− 9.04/16 mm Hg	− 6.8/12 mm Hg
Pivotal/prospective study control arm mean/SD	− 0.74/12 mm Hg	−2.54/16 mm Hg	− 1.8/12 mm Hg
Weibull discount function parameters	Shape: *k* = 3, scale: λ = 0.5	Shape: *k* = 3, scale: λ = 0.5	Shape: *k* = 3, scale: λ = 0.25

*SD* standard deviation, *SE* standard error

### Analysis populations

The intention-to-treat population includes all randomized subjects analyzed according to their randomized treatment group. Safety outcomes, and office and ambulatory blood pressure outcomes will be assessed at each follow-up visit in this population. Subjects who met antihypertensive medication escape criteria will have blood pressure measurements analyzed with LOCF. To apply LOCF, valid office and ambulatory blood pressure measurements taken prior to the subject’s escape date will be used out to 3 months (SPYRAL HTN-OFF MED Pivotal) or 6 months (SPYRAL HTN-ON MED Expansion) in this population. The modified intention-to-treat population does not include subjects who met antihypertensive medication escape criteria. Office and ambulatory blood pressure outcomes will be assessed through 3 months (SPYRAL HTN-OFF MED Pivotal) or 6 months (SPYRAL HTN-ON MED Expansion) in this population. The per protocol population includes the modified intention-to-treat population, but excludes subjects who do not maintain medication adherence as assessed via blood and/or urine testing, and/or have renal anatomy that is ineligible for treatment as assessed by renal angiography and excludes subjects who did not receive their randomized treatment. Office and ambulatory blood pressure outcomes will be assessed through 3 months (SPYRAL HTN-OFF MED Pivotal) or 6 months (SPYRAL HTN-ON MED Expansion) in this population.

#### Subgroup analyses

Subgroup analyses are planned to assess consistency of results between subgroups based on the following demographic or clinical characteristics: gender, age at baseline < 65 vs. ≥ 65 years, BMI by tertiles (kg/m^2^), presence or absence of type 2 diabetes, current smokers vs. non-smokers, baseline eGFR < 60 vs. ≥ 60 mL/min/1.73 m^2^, presence or absence of obstructive sleep apnea, US vs. OUS subjects, US African-American vs. US non-African-American subjects, European vs. Japanese vs. Australian subjects, baseline ambulatory SBP by tertiles and medians (mmHg), baseline office SBP by tertiles and medians (mmHg), baseline ambulatory heart rate by tertiles and medians (bpm), baseline office heart rate by tertiles and medians (bpm), 24 h Pulse Pressure < 60 vs. ≥ 60 mm Hg, presence or absence of orthostatic hypertension at baseline, presence or absence of orthostatic tachycardia at baseline, plasma renin activity at baseline < 0.65 vs. ≥ 0.65 ng/mL/h, aldosterone/renin ratio at baseline by tertiles (ng/dL), aldosterone at baseline by tertiles (ng/dL), tertile analysis by total number of ablations performed, tertile analysis by total number of ablations performed in branch vessels, tertile analysis by total number of ablations performed in main renal artery vessels, tertile analysis by total number of 45 s ablations performed, and medication adherent vs. non-adherent subjects.

### Summaries and comparisons

Continuous outcomes will be summarized with means, standard deviations, medians, and minimums and maximums. Categorical outcomes will be summarized as counts and percentages. Statistical comparisons between treatment groups will be made using *t* tests for continuous outcomes and Chi-square or Fisher’s exact test (depending on overall event rates) for categorical outcomes. Paired *t* tests will be used to compare changes from baseline to follow-up. Additionally, conventional ANCOVA treatment effect estimates will be determined for pivotal/prospective data only and the pooled pivotal/prospective data. A secondary analysis will be performed for both efficacy endpoints where missing outcome data are imputed using a multiple imputation procedure. Missing office and ambulatory primary endpoint outcomes will be imputed using baseline SBP, treatment group, age, gender, and BMI. Unless otherwise specified, a two-sided 0.05 level of significance will be used to determine a significant difference between treatment groups. All non-Bayesian statistical analyses will be performed using SAS for Windows (version 9.2 or higher; SAS Institute, Cary, NC, USA). The Bayesian analysis will be performed using R (version 3.6.0 or higher; The R Foundation, Vienna, Austria) using the bayesDP package [18].

### Analysis of primary safety endpoint

A performance goal approach will be used to analyze the primary safety endpoint. The safety performance goal for major adverse event rate was based on comparison to event rates of other renal interventions [19–27]. The events reported were different among studies; however, for a subset of studies, rates were estimated for a composite of events that was similar to the definition of composite major adverse events in this study. The major adverse event rate from these studies was 7.1%, which will be used as the performance goal for the primary safety endpoint. The primary safety null and alternative hypotheses are *H*_0_: π ≥ 7.1% vs. *H*_a_: π < 7.1%, where π is the major adverse event rate for subjects undergoing renal denervation. Under the assumption that the true rate is 3.5%, and using a one-sided 0.05 level of significance, an evaluable sample size of 253 renal denervation subjects yields 80% power to reject the null hypothesis in favour of the alternative based on a binomial exact test. To account for 5% of subjects lost to follow-up, a total of 266 randomized subjects will be required. Data from the pilot studies, the SPYRAL HTN-OFF MED Pivotal study, the SPYRAL HTN-ON MED Expansion study, and crossovers will comprise the sample cohort.

## Summary

In conclusion, the SPYRAL HTN-OFF MED Pivotal and SPYRAL HTN-ON MED Expansion trials allow a unique opportunity to explore the effect of renal denervation on blood pressure both in the absence of, and presence of, antihypertensive medications, respectively. The trials leverage randomized comparisons to blinded sham controls which allow for an informative, nonbiased approach. The SPYRAL HTN-OFF MED Pivotal and SPYRAL HTN-ON MED Expansion trials have a unique Bayesian design that will exploit historical data from the pilot studies and use a discount function to adjust the influence of the historical data based on level of agreement with data from the prospectively powered studies. There are multiple strengths to using a Bayesian approach in this type of study. First, the ability to leverage information from a prior trial of similar design increases the power of the current trial, and conversely, decreases the overall number of subjects required for randomization in the current trial. Second, the adaptive nature of a Bayesian design supports interim looks with stopping for efficacy or futility. Finally, a novel Bayesian approach makes efficient use of prior information and further advances a development program by seamlessly combining trials of a similar clinical design.

## Electronic supplementary material

Below is the link to the electronic supplementary material.

Supplementary file1 (DOCX 33 kb)

## Abbreviations

ABPM: Ambulatory blood pressure monitor
CEC: Clinical Events Committee
DBP: Diastolic blood pressure
DSMB: Data Safety Monitoring Board
eGFR: Estimated glomerular filtration rate
SBP: Systolic blood pressure
