# Correction to: Rationale and design of two randomized sham‑controlled of catheter‑based renal denervation in subjects with uncontrolled hypertension in the absence (SPYRAL HTN‑OFF MED Pivotal) and presence (SPYRAL HTN‑ON MED Expansion) of antihypertensive medications: a novel approach using Bayesian design

**DOI:** 10.1007/s00392-020-01620-1

**Published:** 2020-04-08

**Authors:** Michael Böhm, Raymond R. Townsend, Kazuomi Kario, David Kandzari, Felix Mahfoud, Michael A. Weber, Roland E. Schmieder, Konstantinos Tsioufis, Graeme L. Hickey, Martin Fahy, Vanessa DeBruin, Sandeep Brar, Stuart Pocock

**Affiliations:** 1 Universitätsklinikum des Saarlandes, Saarland University, Homburg, Germany; 2 Perelman School of Medicine, University of Pennsylvania, Philadelphia, PA USA; 3 Jichi Medical University, Tochigi, Japan; 4 Piedmont Heart Institute, Atlanta, GA USA; 5 State University of New York, Downstate Medical Center, Brooklyn, NY USA; 6 University Hospital Erlangen, Erlangen, Germany; 7 National and Kapodistrian University of Athens, Athens, Greece; 8 Medtronic PLC, Santa Rosa, CA USA; 9 London School of Hygiene and Tropical Medicine, London, UK

## Correction to: Clinical Research in Cardiology (2020) 109:289–302 https://doi.org/10.1007/s00392-020-01595-z

The original version of this article unfortunately contained a mistake. The sentence within the section “Bayesian design and prespecified interim analyses” should read:

Due to a Bayesian power prior approach being used, a non-standard parameterization for the ANCOVA model is used to allow for informative prior distributions to be placed separately on the RDN and control arm effects:

$${y}_{i}= {\mu }_{t}I\left(i\in t\right)+{\mu }_{c}I\left(i\in c\right)+{x}_{i}\beta +{\varepsilon }_{i}; {\varepsilon }_{i} \sim N\left(0, {\sigma }^{2}\right),$$

where ${y}_{i}$ denotes the change from baseline in BP at follow-up, subscripts $i$ denote the $i$th subject with evaluable data, $I\left(i\in t\right)=1$ if subject $i$ is randomized to renal denervation, or $=0$ if randomized to sham control, and $\beta$ is the regression coefficient for the adjustment in mean-centered baseline BP, ${x}_{i}$.

The original article has been corrected.

