# Supplementary material for: Rationale and design of two randomized sham-controlled trials of catheter-based renal denervation in subjects with uncontrolled hypertension in the absence (SPYRAL HTN-OFF MED Pivotal) and presence (SPYRAL HTN-ON MED Expansion) of antihypertensive medications: a novel approach using Bayesian design
Source: Clin Res Cardiol. 2020 Feb 7;109(3):289–302. doi: 10.1007/s00392-020-01595-z (PMC7042193; doi:10.1007/s00392-020-01595-z)
Supplement: Supplementary file 1 — Supplementary file1 (DOCX 33 kb) [file 392_2020_1595_MOESM1_ESM.docx]

Supplement to

Rationale and design of two randomized sham-controlled trials of catheter-based renal denervation in patients with uncontrolled hypertension in the absence (SPYRAL HTN-OFF MED) and presence (SPYRAL HTN-ON MED) of antihypertensive medications: a novel approach using Bayesian design

Michael Böhm, Raymond R. Townsend, Kazuomi Kario, David Kandzari, Felix Mahfoud, Michael A. Weber, Roland E. Schmieder, Konstantinos Tsioufis, Graeme L. Hickey, Martin Fahy, Vanessa DeBruin, Sandeep Brar, Stuart Pocock

Contents

Blood Pressure Measurement……………………………………………………………………………..………….3

Office Blood Pressure……………………………………………………………………………….………..3

Ambulatory Blood Pressure Monitoring………………………………………………………….….7

Medication Re-Introduction…………………………………………………………………………..……………….9

**Supplemental Methods**

**Blood Pressure Measurement Procedures**

OFFICE BLOOD PRESSURE

All Office Blood Pressure (OBP) Measurements must be taken with the automatic BP

Monitor & printer as specified by the sponsor.

- At screening visit 1, the appropriate arm for study measures must be selected as specified in section A below and then used for all subsequent follow-up visits.
- For each study visit, the study visit should begin before 10:30am. This does not apply to Unscheduled follow-up visits.
- Patient should not take their antihypertensive medication in the morning of the visit, but rather bring the medication with them to the visit to have observed pill taking after office blood pressure measurement (if applicable). This does not apply to the medication reintroduction visit or at the discharge visit.

ARM SELECTION AT SCREENING VISIT 1 ONLY

1. If the subject is on anti-hypertensive medications, the visit should begin before 10:30am unless the subject normally takes their anti-hypertensive medication in the afternoon in which the subject’s visit can occur in the afternoon.
2. With subject prepped per “Preparation” section below, measure BP in each arm. Ensure each measurement is captured/recorded and identifies on which arm the BP was measured.
3. Use the arm with the higher systolic BP for screening measurements and all subsequent measurements
   - If there is a reason to use a particular arm, document the reason and use that arm for all measures going forward.

**PREPARATION AT ALL VISITS**

1. Ensure the BP monitor and all necessary equipment are functioning appropriately (per sponsor instructions).
2. Confirm the subject did not drink coffee or alcohol, smoke, or exercise within 30 minutes prior to the measurements.
3. Request the subject to use the bathroom prior to measurements (a full bladder can affect the reading).
4. The subject should be seated comfortably with the back supported and the upper arm bared with no clothing between the arm and BP cuff. The legs should not be crossed.
5. Ensure that the BP cuff is appropriately sized (see Table 14 below) and that the upper arm is supported at the level of the heart (e.g. resting on a table at the level of his/her heart).

**BP Cuff Size Chart**

| **Cuff Size*** | **Fits Arm Circumference of (inches)** | **Fits Arm Circumference of (centimeters)** |
| --- | --- | --- |
| **Small** | 7-9 | 17-22 |
| **Medium** | 9-13 | 22-32 |
| **Large** | 13-17 | 32-42 |
| **Extra-Large **** | 17-20 | 42-50 |

* If a subject is on the border of two cuff sizes, opt for the larger of the two sizes.

** Subjects requiring greater than an extra-large cuff size at time of screening must be excluded from the study

1. Perform a “test” BP measure. Ensure test measurement is captured/recorded.
2. Have the subject sit comfortably and quietly for at least 5 minutes, but no more than 10 minutes, with back supported and feet flat on the ground (i.e., not on an exam table, legs not crossed)

**METHOD FOR TAKING BP AT ALL VISITS**

1. General Instructions
   1. With subject prepared per “Preparation” section above and using arm selected at Screening, take at least three (3) seated BP measurements in order to obtain the BP average.
   2. Wait at least 1 minute between each measurement. **Ensure that the blood pressure monitor time clock is used for tracking the time intervals to avoid deviations due to insufficient wait time between measurements.**
   3. Print (if available) and label after each measurement.
2. Three **(3) consecutive, consistent seated** BP measurements must be used to obtain the BP average.
   1. If the lowest and highest systolic BP (SBP) values of the first 3 consecutive measurements are >15 mmHg apart, take one additional reading and average the last 3 consecutive measurements (measurements 2-4). If the measurements are still >15 mmHg apart, take one additional reading and average the last 3 consecutive measurements (measurements 3-5). If the measurements are still >15 mm Hg apart, take one final measurement and average the last 3 consecutive measurements (measurements 4-6).
3. **At Screening Visit:** If the lowest and highest SBP values for the readings are more than 20 mm Hg apart after 6 measurements, the subject must be excluded from the study.
4. **At all Subsequent Follow Up Visits:** If the lowest and highest SBP values for the readings are more than 20 mm Hg apart after 6 measurements, take the average of the last three measurements (measurements 4 – 6) and record the value on the CRF.
5. Record the **last** 3 consecutive, consistent readings on the CRF (i.e. cannot pick the ‘best’ 3).

| ***NOTE:*** *To better ensure long-term preservation of the OBP source data, a photocopy labeled as certified of all automatic BP Monitor print-outs should be made and attached to the originals. If unable to print, document BP & HR values, dates and exact times of readings, and label appropriately.* |
| --- |
| Orthostatic Hypotension Evaluation (AT SCREENING VISIT 2 ONLY) |

In addition to the seated OBP recordings above, measure supine and standing BPs.

1. Have the subject lie supine for at least 5 minutes prior to taking the supine BP measurement.
2. Measure BP within 1-3 minutes upon standing for the standing measurement. Standing must follow the supine to measure orthostatic effect.
   - Evaluate for any symptoms (e.g., dizziness) that may occur in the subject within the first 3 minutes after standing.

AMBULATORY BLOOD PRESSURE MONITORING

All 24-hour Ambulatory Blood Pressure Monitoring (ABPM) measurements must be taken with the 24-hour ABPM device provided by the sponsor to ensure consistency.

1. If the subject is on anti-hypertensive medications, the visit should begin before 10:30am unless the subject normally takes their anti-hypertensive medication in the afternoon in which the subject’s visit can occur in the afternoon.
2. Study personnel should observe the subject swallowing the antihypertensive medication(s), if applicable. Once this is completed and documented, the ABPM device should be applied to the subject and the recording started before leaving the office.
3. Place cuff on the subject’s non-dominant arm.
4. Instruct the subject in proper cuff positioning in case they must remove it but stress the importance of leaving the BP cuff on.
5. The ABPM has pre-set parameters and should not be adjusted. These parameters are set to record blood pressure every 30 minutes.
6. Instruct subjects that they should engage in their usual physical level but should avoid strenuous exercise during the monitoring period.
7. Instruct the subject to hold the arm still by the side while the device is taking a reading.
8. Upon the return of the ABPM machine:

- Submit the 24-hour ABPM data to Medtronic
- A 24-hour ABPM will be considered adequate if the number of successful daytime readings captured is ≥ 21 and the number of successful nighttime readings captured is ≥ 12. If the minimum number of readings was not reached at Screening Visit 2 and it was not due to a technical failure, the subject will be considered a screen failure. For all other time points with ABPM, make all efforts to obtain repeat ABPM from subject until the minimum number of readings is obtained. At Screening Visit 2, a single repeat ABPM will be allowed in the event of technical issues or if the minimum required number of daytime and nighttime readings is not obtained.

**Medication Re-Introduction for Subjects with 3-Month Systolic Office Blood Pressure ≥140 mm Hg**

Upon completion of a valid ABPM at the 3-month follow-up visit, subjects with an office

SBP<140 mmHg do not need to be followed up for study purposes until the 6 month follow-up

visit. Subjects with an office SBP ≥ 140 mmHg will begin an antihypertensive medication

regimen of one or more of the following classes individually or in a combination pill with dosing

at the discretion of the study investigator:

- ACE/ARB
- Calcium Channel Blocker
- Thiazide-Type Diuretic

Subjects will return at 4 months (± 7 days) to perform a clinical assessment and obtain an

Office Blood Pressure according to the guidelines on Blood Pressure Measurement Procedures.

- The investigator may utilize his or her discretion in modifying the antihypertensive regimen.
- Any modification to the anti-hypertensive medication regimen must be documented on the eCRFs.

To provide the appropriate oversight for study subjects, the visit schedule between the required 4-month and 6-month visits may need to be adapted to account for a subject's clinical status, complexity of the medication regimen, and/or the subject’s blood pressure. If additional changes to the anti-hypertensive medication regimen are made between 3 and up to 6 months post-procedure, complete an unscheduled follow-up visit, and obtain an Office Blood Pressure according to the guidelines on Blood Pressure Measurement Procedures. In addition, all medication changes are to be documented on the eCRF. All medication used should be commercially available in the respective geographies and compliant with local labeling.
